# Healthcare practices that increase the quality of care in cancer trajectories from a general practice perspective: a scoping review

**DOI:** 10.1080/02813432.2022.2036421

**Published:** 2022-03-07

**Authors:** Anne Nicolaisen, Gitte Bruun Lauridsen, Peter Haastrup, Dorte Gilså Hansen, Dorte Ejg Jarbøl

**Affiliations:** a Research Unit for General Practice, Department of Public Health, University of Southern Denmark, Odense C, Denmark; b Center for Shared Decision Making, Lillebaelt Hospital, University Hospital of Southern Denmark, Vejle, Denmark; c The Department of Regional Health Research, University of Southern Denmark, Odense C, Denmark

**Keywords:** Cancer, patient care management, patient-focused care, physician-patient relation, caregivers

## Abstract

**Objective:**

General practice plays an important role in cancer trajectories, and cancer patients request the continuous involvement of general practice. The objective of this scoping review was to identify healthcare practices that increase the quality of care in cancer trajectories from a general practice perspective.

**Design, setting, and subjects:**

A scoping review of the literature published in Danish or English from 2010 to 2020 was conducted. Data was collected using identified keywords and indexed terms in several databases (PubMed, MEDLINE, EBSCO CINAHL, Scopus, and ProQuest), contacting key experts, searching through reference lists, and reports from selected health political, research- and interest organizations’ websites.

**Main outcome measures:**

We identified healthcare practices in cancer trajectories that increase quality care. Identified healthcare practices were grouped into four contextual domains and allocated to defined phases in the cancer trajectory. The results are presented according to the Preferred Reporting Items for Systematic Reviews and Meta-analysis extension for scoping reviews (PRISMA-ScR).

**Results:**

A total of 45 peer-reviewed and six non-peer-reviewed articles and reports were included. Quality of care increases in all phases of the cancer trajectory when GPs listen carefully to the full story and use action plans. After diagnosis, quality of care increases when GPs and practice staff have a proactive care approach, act as interpreters of diagnosis, treatment options, and its consequences, and engage in care coordination with specialists in secondary care involving the patient.

**Conclusion:**

This scoping review identified healthcare practices that increase the quality of care in cancer trajectories from a general practice perspective. The results support general practice in investigating own healthcare practices and identifying possibilities for quality improvement.

**KEY POINTS**
- Identified healthcare practices in general practice that increase the quality of care in cancer trajectories:
- Listen carefully to the full story
- Use action plans and time-out-consultations
- Plan and provide proactive care
- Act as an interpreter of diagnosis, treatment options, and its consequences for the patient
- Coordinate care with specialists, patients, and caregivers with mutual respect
- Identified barriers for quality of care in cancer trajectories are:
- Time constraints in consultations
- Limited accessibility for patients and caregivers
- Health practices to increase the quality of care should be effective, safe, people-centered, timely, equitable, integrated, and efficient. These distinctions of quality of care, support general practice in investigating and improving quality of care in cancer trajectories.

## Background

General practice has a significant role in cancer patients’ trajectories because general practice is often the patients’ first contact with the healthcare system [1]. The implementation of cancer pathways and fast-track referral pathways has reduced the time to diagnosis and treatment [2,3]. However, signs of cancer are intangible and only half of the patients, who were later diagnosed with cancer, presented to their general practitioner (GP) with cancer alarm symptoms [4]. An increased number of GP visits often precedes cancer diagnosis [5], but pre-diagnostic healthcare-seeking varies greatly among patients with different cancer types and socioeconomic status [6,7]. Even though the incidence of cancer is increasing with the growing and ageing population; typically, a GP only has a few cases a year where patients are diagnosed with cancer [1,8]. The incidence of cancer is steadily increasing globally, with high-income countries accounting for the main proportion [9]. However, the incidence of different types of cancer differs greatly and presentations vary, making it difficult for GPs to recognize diagnostic patterns for specific cancer types.

A review found that cancer patients preferred their GP to be continuously involved in the cancer trajectory [10]. Due to being based in local communities and providing person-centered care, general practice is the obvious choice for cancer patients’ follow-up consultations [1,11]. GPs expressed the need for more specific information regarding their patients diagnosed with cancer, from secondary healthcare at the transition of care to primary care, including the possibility of coordinating with and counseling from cancer specialists at hospitals [10,12,13]. Also, cancer patients felt more secure and were more satisfied with follow-up in general practice, if the GP had the possibility of counseling with a specialist [14]. However, there are still barriers to coordinating care, such as defining and agreeing on the health professionals’ roles and responsibilities, lack of coordinating the transition of care, and inadequate communication between cancer/hospital specialists and general practice [15].

In general, there are many evidence-based guidelines and scientific reviews that recommend predefined and standardized processes of care for cancer trajectories, making it difficult for GPs to be updated on all of them [16]. Moreover, evidence-based guidelines are not directly transferable to primary care due to the individual contextual factors in each patient-GP relation, e.g. comorbidities, sex, age, social, economic, cultural, and occupational factors [17]. To our knowledge, no reviews investigated how evidence-based guidelines and standards are translated into healthcare practices in general practice, in which they took contextual factors into account. This scoping review aims to identify healthcare practices that increase the quality of care in cancer trajectories, from a general practice perspective.

## Materials and methods

The scoping review methodology was chosen for this study, as it goes beyond effectiveness by investigating both the context in which care is delivered and the knowledge gaps [18,19]. Joanna Briggs Institute methodology [19] was used for the search strategy (Appendix I), and the search results are presented according to the Preferred Reporting Items for Systematic Reviews and Meta-analysis extension for scoping reviews (PRISMA-ScR) [20].

### Framework for cancer trajectories

Based on the current literature, seven phases of cancer trajectories were defined; 1. Awareness of patients’ bodily sensations and unexplained symptoms, 2. First presentation and investigation of symptoms in primary care, 3. Referral to secondary care, 4. Diagnosis, 5. Treatment, 6. Follow-up, and 7. Palliative care. More Specifically, phase 1–4 are based on The Aarhus Statement [21], 5 and 6 are based on the ‘Quality of Cancer Survivorship Care Framework’ [22], and phase 7 is based on the WHO report: ‘Integrating palliative care and symptom relief into primary health care’ [23]. After being diagnosed with cancer, patients may interchangeably receive treatment, follow-up, and/or palliative care; therefore, the phases should be regarded as concurrent and not sequentially.

### Eligibility criteria

Studies describing healthcare practices in cancer trajectories from a general practice (i.e. primary healthcare, family medicine, GPs, and general practice staff) perspective included: patients diagnosed with cancer—regardless of age, type or stage of cancer, patients with unexplained or cancer suspicious symptoms, and caregivers of these patient groups. Furthermore, studies with both GPs and staff/health personnel employed in general practice were included. Patients and caregivers are age 18+ unless referred to as otherwise. Studies were excluded if they were solely concerned: pharmacies, nursing homes, community nurses, or private practice specialists, such as gynecologists.

#### Theoretical frameworks

The WHO’s definition of healthcare quality [24] was used to define and categorize how healthcare practices affect the quality of care in cancer trajectories and identify which healthcare practices increase the quality of care (Table 1).

**Table 1.** Definition of quality healthcare based on a summary of a selection of the main components of definitions of quality healthcare in the reference [24].

Definition of quality healthcare
In accordance with the WHO, quality healthcare should be:
Effective: providing evidence-based healthcare services to those who need them Safe: avoiding harm to people for whom the care is intended People-centred: providing care that responds to individual preferences, needs and values Timely: reducing waiting times and sometimes harmful delays for those who both receive and give care Equitable: providing care that does not vary in quality on account of age, sex, gender, race, ethnicity, geographical location, religion, socioeconomic status, linguistic or political affiliation Integrated: providing care that is coordinated across levels and providers, and makes available the full range of healthcare services throughout the life course Efficient: maximizing the benefit of available resources and avoiding waste

We used the four contextual domains included in the Quality of Cancer Survivorship Framework to group healthcare practices according to how they are affected by or how they affected the context of cancer trajectories in general practice [22]; i.e. I. Clinical structure, II. Communication/decision making, III. Care coordination, and IV. Patient/caregiver experiences.

### Search strategy

The search strategy in scoping reviews implies an iterative search technique and is based on both a systematic scoping search of peer-reviewed literature and a screening of non-peer-reviewed literature from January 2010 to September 2020. The systematic search included peer-reviewed literature with any study design and methodology, written in English or Danish. Included studies described healthcare practices in terms of, i.e. testing interventions or everyday experiences/healthcare practices in cancer trajectories. Studies that included expectations, views, and beliefs as their findings, were excluded. Reference lists of the included articles for full-text reading were screened for relevant articles for full-text reading, and experts within the field of general practice and cancer trajectories were consulted to identify further relevant peer-reviewed and non-peer-reviewed records.

An initial search for identified keywords and index terms was conducted in PubMed, and a second search was conducted in MEDLINE, EBSCO CINAHL, Scopus, and ProQuest. A research librarian assisted with the second search that was conducted in March 2020 and updated in September 2020. The search strategy used in MEDLINE is presented in Appendix I. All identified records were imported to the web-based screening software, Covidence (www.covidence.org), and duplicates were removed. Two reviewers screened titles and abstracts (AN and GBL). The screening was pilot tested on 25 articles before the reviewers screened them independently. The same reviewers also conducted the full-text screening, which was pilot tested on a random sample of five articles. Disagreements were solved through discussion until consensus was reached.

Records identified through other sources were non-indexed reports, government documents, guidelines, and newsletters relevant to general practice. Other sources included these Danish websites: The Danish Cancer Society, Danish Health Authority, The Danish Knowledge Centre for Rehabilitation and Palliative Care (REHPA), The Danish College of General Practitioners (DSAM), Monthly Magazine for General Practice [Månedsskrift for Almen Praksis], and corresponding websites in the UK (e.g. The Kings Fund and United Kingdom National Health Service). Non-peer reviewed records were gathered from the UK, as the UK has a primary healthcare system comparable to Denmark and has experienced similar challenges regarding cancer trajectories as Denmark.

### Synthesis of results

An interpretive approach was applied to identify healthcare practices in the included records and was performed by AN and GBL. The identified healthcare practices were grouped into the corresponding contextual domain(s) for each of the seven cancer trajectory phases and assigned by their effect on the quality of care according to the WHO definition.

## Results

**Figure 1.**
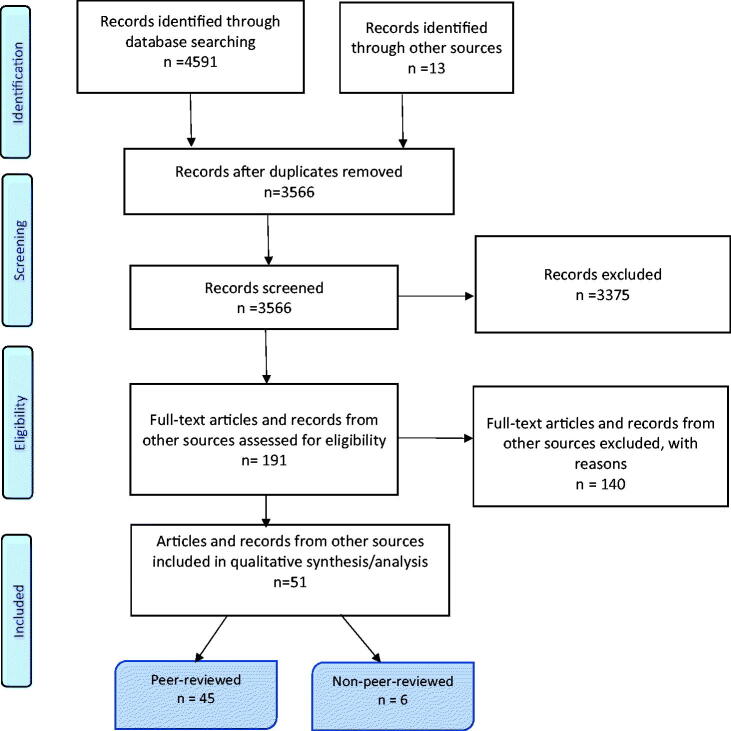
PRISMA-ScR flowchart presenting the study selection process in the systematic scoping review.

A total of 3553 articles were screened for eligibility, and 178 peer-reviewed full-text articles were retrieved and reviewed. The main reason for exclusion was that no healthcare practices were described (*n* = 102). A total of 45 peer-reviewed and six non-peer-reviewed articles were included (Table 2). The study’s selection process is presented in Figure 1. Overall, we identified five healthcare practices that increased the quality of care in cancer trajectories from a general practice perspective (Table 3). In the following text, quality concepts corresponding to the WHO definition are highlighted in *italics.*

**Table 2.** Study characteristics organized by study methodology and cancer trajectory phase.

Authors (year), country	Trajectory phase(s)	Participant and data characteristics	Aim of the article
Intervention studies			
Grange et al. (2014), France [29]	2. First presentation and investigation of symptoms	Self-reported questionnaires from 364 GPs at baseline	To investigate the efficacy of a general practitioner awareness and training campaign compared for improving early diagnosis of melanoma
Toftegaard et al. (2016), Denmark [30]	2. First presentation and investigation of symptoms 3. Referral to secondary care	Self-reported questionnaires from 202 GPs at baseline Self-completed forms for cancer risk-assessment from 532 GPs before and 524 GPs after continuing medical education (CME)	To investigate the effect of standardized CME aimed to optimize cancer-related referrals from general practice to hospitals by reducing the GPs’ referral threshold and to increase their knowledge about cancer symptoms to identify underlying cancers at an earlier stage
Wieldraaijer et al. (2019), Netherlands [75]	4. Diagnosis 5. Treatment	Self-reported questionnaires from 170 colorectal cancer patients (72 patients before and 98 patients after the introduction)	To investigate the effect of introducing “time out consultations” (TOC) with GPs between diagnosis and active treatment, on change in number, kind, and content of consultations, and in patient-reported outcomes
Bergholdt et al. (2013a), Denmark [57]	5. Treatment 6. Follow-up	Self-reported questionnaires from 612 newly diagnosed cancer patients at baseline (intervention group *n* = 296, control group *n* = 316) and 776 GPs (intervention group *n* = 399, control group *n* = 377)	To evaluate the effects of an intervention encouraging early involvement of GPs in cancer rehabilitation assessed on satisfaction of patients with their GP in general, in relation to the cancer course, and in GPs’ self-reported satisfaction with their own contribution to their patients’ physical and psychosocial rehabilitation.
Bergholdt et al. (2013 b), Denmark [66]	5. Treatment 6. Follow-up	Self-reported questionnaires from 612 newly diagnosed cancer patients at baseline (intervention group *n* = 296, control group *n* = 316) and 752 GPs (intervention group *n* = 373, control group *n* = 379)	To evaluate effects of the intervention encouraging GPs to proactively contacting patients, on patients’ participation in rehabilitation activities, and on whether proactivity is associated with patients’ participation in rehabilitation
Bergholdt et al. (2012), Denmark [65]	5. Treatment 6. Follow-up	Self-reported questionnaires from 612 newly diagnosed cancer patients at baseline (intervention group *n* = 296, control group *n* = 316) and 752 GPs (intervention group *n* = 373, control group *n* = 379)	To investigate the effect of an intervention giving the GP an enhanced role in improving patients’ health-related quality of life and psychological distress following cancer
Boekhout et al. (2015), Canada [68]	5. Treatment 6. Follow-up	Self-reported questionnaires from 337 breast cancer patients at baseline (Intervention *n* = 164, Control *n* = 173)	To investigate the effect of implementing a shared care plan in the transition of survivorship care from specialists to primary care physicians (PCP) on health service outcomes and patient-reported outcomes
Fairweather et al. (2020), Australia [55]	5. Treatment 6. Follow-up 7. Palliative care	Self-reported questionnaires from 35 GPs and 17 hospital staff	To improve two-way communication between hospital- and community-based care providers by having two GPs from the local community attending multi-disciplinary meetings in a liaison role as a primary care representative
Stegmann et al. (2020) Netherlands [53]	5. Treatment	Self-reported questionnaires from 114 older patients with non-curable cancer at baseline (Intervention group *n* = 53, control group *n* = 61)	To assess the utility of the Outcome Prioritisation Tool (OPT), designed to aid GPs discussion with a patient about treatment goals and to empower patients
Trabjerg et al. (2020 b) Denmark [48]	5. Treatment 6. Follow-up 7. Palliative care	Self-reported questionnaires from 44 cancer patients at baseline assigned to the intervention group (breast *n* = 6, lung *n* = 17, colorectal *n* = 15, others *n* = 6), 39 GPs and 15 oncologists	To analyze video consultations (The Partnership Intervention) from a user perspective (patients, GPs and oncologists), based on three surveys of patients enrolled in the intervention group, their oncologists, and GPs
Pelayo-Alvarez et al. (2013), Spain [73]	7. Palliative care	Self-reported questionnaires from 117 patients with advanced cancer (10 different cancer types) at baseline (Intervention *n* = 63, Control *n* = 54), 84 caregivers at baseline (Intervention *n* = 48, Control *n* = 36) and 145 PCPs at baseline	To investigate the effect of an online education model for palliative care (PC) targeted to primary care physicians (PCPs) on symptom control, quality of Life (QOL), main caregiver satisfaction, PCP’s level of knowledge, and PCP’s attitude towards PC and satisfaction.
Interview and observation studies			
Bergin et al. (2020), Australia [27]	1. Awareness of patients’ bodily sensations and unexplained symptoms 2. First presentation and investigation of symptoms 4. Diagnosis	43 interviews with cancer patients post diagnosis Colorectal cancer (*n* = 21) Breast cancer (*n* = 22) Supplemented with information from a patient, Primary Care Practitioner (PCP), and specialist survey	To explore how and why cancer pathways may differ by residential location for colorectal and breast cancers with different rural-urban disparity profiles
Almuammar (2020), Saudi Arabia [31]	2. First presentation and investigation of symptoms	Interviews with 20 patients with common cancer diagnosis, and 15 GPs	To investigate factors that contribute to ‘late-stage presentation’ of common cancers at cancer centers from a patient and GP perspective
Amelung et al. (2020), England [34]	2. First investigation and presentation of symptoms	Qualitative analysis of 80 video-consultations. 20 interviews with patients presenting a new or persistent problem in general practice, and 7 GP interviews representing 7 general practices	To understand doctor-patient communication around the significance of persistent or new presenting problems and its potential impact on timely cancer diagnosis
Brindle et al. (2012), England [25]	2. First presentation and investigation of symptoms	Interviews with 22 patients with early-stage lung cancer	To investigate why symptoms indicative of early-stage lung cancer were not presented to general practitioners and how early symptoms might be better elicited within primary care
Clarke et al. (2014), England [28]	2. First presentation and investigation of symptoms	Interviews with 18 mothers and 3 fathers of 18 children with a diagnosis of acute leukemia, and 9 GP’s	To investigate the prehospital presentation of pediatric leukemia and identify the disease and non-disease related factors which facilitate or impede diagnosis
Evans et al. (2019), UK [35]	2. First presentation and investigation of symptoms	Interviews with 23 cancer patients and 25 GPs	To explore patients’ and GP’s accounts of how responsibility for follow-up was perceived and shared in their experiences of cancer safety netting occurring within the past 6 months
Hultstrand et al. (2020a), Sweden [26]	2. First presentation and investigation of symptoms	Observation of 18 consultations with patients seeking care for sensations/symptoms that could indicate cancer, or had worries about cancer and the involved GP	To explore how presentations of bodily sensations were constructed and legitimized in primary care encounters within the context of Standard Cancer Patient Pathways
Hultstrand et al. (2020 b), Sweden [33]	2. First presentation and investigation of symptoms	13 interviews with GPs at 4 primary healthcare centers	To explore how GPs assign meanings and act upon patients’ symptoms in primary care encounters in the context of standardized cancer patient pathways
DiCicco-Bloom et al. (2013), USA [47]	2. First presentation and investigation of symptoms 4. Diagnosis 5. Treatment 6. Follow-up	Interviews with 11 primary care physicians (PCPs) and 10 nurse practitioners	To provide a better understanding of the nature of interactions among PCPs, patients, and oncologists throughout the cancer care continuum, to better understand the transition to survivorship
Piano et al. (2019), UK [38]	3. Referral to secondary care	Four focus groups with 29 patients who have completed diagnostic tests and received a non-malign test result within the last 6 months	To explore public attitudes towards the Faster Diagnosis Standard (FDS) within the context of recent referral experiences
Dahlhaus et al. (2014), Germany [41]	4. Diagnosis 5. Treatment 6. Follow-up	Interviews with 30 GPs	To provide a better understanding of the nature of interactions among primary care clinicians, patients, and oncologists throughout the cancer care continuum to better understand the transition to survivorship
Coindard et al. (2016), France [50]	5. Treatment	Interviews with 50 cancer patients Breast cancer (*n* = 20) Colorectal cancer (*n* = 14) Lung cancer (*n* = 9) Prostate cancer (*n* = 7)	To investigate if and why cancer patients consult their GP during the initial phase with intravenous chemotherapy, and assessment of their GP’s role in their treatment
Brandenbarg et al. (2016), Netherlands [49]	5. Treatment 6. Follow-up	Interviews with 22 cancer patients Colon cancer (*n* = 14) Rectum cancer (*n* = 8)	To clarify experiences and preferences of patients regarding the current and future role of general practitioners during treatment and follow-up care of colorectal cancer
Burridge et al. (2011), Australia [44]	5. Treatment 6. Follow-up 7. Palliative care	Interviews with 6 lay cancer caregivers and 19 health professionals Practicing GP (*n* = 6), palliative specialist (*n* = 5), oncologist (*n* = 2), caregiver representatives (*n* = 3), and other (*n* = 3)	To examine what the views of lay caregivers and health professionals reveal about the way lay caregivers’ health concerns are raised with their GP
Hall et al. (2012), UK/Scotland [54]	5. Treatment 6. Follow-up	Interviews in year 2002: 39 colorectal cancer (CRC) patients Interviews in year 2009: 30 CRC patients	To explore experiences and support needs of people with CRC To identify opportunities for supportive primary care interventions with potential to benefit people with CRC
Browne et al. (2011), Scotland [42]	5. Treatment 6. Follow-up	Interviews with 24 newly diagnosed colorectal cancer patients (15 female/9 males age range 34–84 years)	To explore colorectal cancer patients’ experiences of psychosocial problems and their management in primary and specialist care
Adams et al. (2011), England [36]	6. Follow-up	Interviews with 38 patients with 12 different cancer types Six focus groups with primary care teams from 6 practices (31 GPs, 1 GP trainee, 1 medical student, 13 practice nurses, 2 district nurses, and 23 other healthcare professionals)	To implement the Quality and Outcomes Framework (QOF) cancer care review, and to investigate: patients’ experiences of primary care over the first 3 years following a cancer diagnosis, patients’ views on optimal care, and views of primary care professionals regarding cancer care
Bowmann et al. 2010, USA [67]	6. Follow-up	Interviews with 215 older long-term cancer survivors Breast cancer (49.8%) Colorectal cancer (24.7%) Prostate cancer (25.6%)	To investigate cancer survivors’ reports of primary care physicians (PCP) involvement in; discussing cancer history, whether the PCP initiated discussions, and whether discussions led to tests/procedures
Geelen et al. (2014), Netherlands [62]	6. Follow-up	Interviews with 35 primary care professionals (11 GPs)	To explore how a proactive and holistic approach in cancer survivorship care fit in with ‘habitus’ and everyday practice of GPs
Margariti et al. (2020), England [58]	6. Follow-up	Semi-structured telephone interviews with 20 GPs	To examine the preparedness, concerns, and experiences of GPs in relation to their role in providing follow-up care to prostate cancer survivors
Murchie et al. (2010), Scotland [39]	6. Follow-up	Interviews with 18 patients with cutaneous melanoma, purposely sampled from the intervention group in an RCT study assessing the effect of GP-led melanoma follow-up	To explore patient’s practical experiences and feelings about receiving structured melanoma follow-up from their GP
van Leeuwen et al. (2018), Netherlands [60]	6. Follow-up	Interviews with 10 GPs participating in a project where GPs were responsible for complete survivorship care including recurrence detections and rehabilitation	To evaluate the experiences of GPs with monthly oncology meetings in a GP-practice to support GP-led survivorship care of colon cancer patients
Waterland et al. (2020), Australia [64]	6. Follow-up 7. Palliative care	Interviews with 23 GPs	To report GPs’ experiences of providing nutrition and exercise advice to their patients, and to identify perceived barriers and enablers to further implementation of exercise and nutrition advice throughout the cancer journey from a GP perspective
Beernaert et al. (2014), Belgium [46]	7. Palliative care	Interviews with 18 patients: Cancer (*n* = 6), COPD (*n* = 3), Heart failure (*n* = 3), Dementia (*n* = 6) Six focus groups with family physicians (*n* = 20) and community nurses (*n* = 12)	To explore the barriers to and facilitators of the early identification by family physicians of the palliative care needs
Couchman et al. (2020), England [74]	7. Palliative care	Interviews with 15 cancer patients	To explore patients’ views and experiences of: The role of the family physician (FP) in providing palliative care to adult patients with cancer, and the facilitators and barriers to the FP’s ability to fulfil this perceived role
van Gurp et al. (2016), Netherlands [59]	7. Palliative care	Observation of 129 teleconsultations with 18 palliative care patients (*n* = 16 cancer patients, 17 informal caregivers, 15 primary care physicians (PCPs) and 12 specialist palliative care team clinicians (SPCT) Interviews with 9 patients, 9 informal caregivers, 14 PCPs and 1 homecare nurse	To explore whether and how teleconsultation supports the integration of primary care, specialist palliative care, and patient perspectives and services How patients and (in)formal caregivers experience collaboration in a teleconsultation approach
Other peer-reviewed studies			
Fraulob et al. (2020) England [32]	2. First presentation and investigation of symptoms 3. Referral to secondary care 4. Diagnosis 5. Treatment 6. Follow-up	84 specific comments about general practice in a qualitative survey feedback from brain cancer patients	To understand patients’ experiences of general practice care in more detail by identifying the range of issues described in comments and to use these analyses to suggest ways in which care and support may be improved.
Noteboom et al. (2020), Netherlands [51]	4. Diagnosis 5. Treatment	Patients with a new diagnosis of metastatic gastrointestinal or lung cancer, or having changes in treatment perspective Self-reported questionnaires from patients (*n* = 12), GPs (*n* = 18), specialist (*n* = 8) Semi-structured interviews: 9 patients and 5 GPs	To explore uptake and first experiences with a Time Out Consultation (TOC) concerning experienced added value for Shared Decision Making (SDM) according to patients, family physicians and specialists
Trabjerg et al. (2020a), Denmark [52]	5. Treatment 6. Follow-up 7. Palliative care	Recordings of 12 video consultations with 12 cancer patients (colorectal *n* = 2, lung *n* = 6, gynecological *n* = 1, breast *n* = 1, pancreatic *n* = 2), 8 oncologists, and 11 GPs	To explore the consultation structure, health concerns, and patient-centeredness when two doctors are attending consultations through video with a patient with cancer at the offices of oncologists or GPs
Collie et al. (2014), Canada [56]	6. Follow-up	Survey of 54 cancer survivors (Head-and-neck and breast cancer), 22 family physicians, and 9 nurses Interviews with cancer survivors and healthcare professionals Head-and-neck cancer (*n* = 4), Breast cancer (*n* = 8) Family physicians (*n* = 3), Specialist nurses (*n* = 9)	To assess the value of survivorship care plans for cancer survivors
Nababan et al. (2020), Australia [63]	6. Follow-up	Questionnaires: 75 lung cancer patients Interviews: 47 lung cancer patients	To assess patients’ experience of GP involvement following lung cancer diagnosis, and patients’ view on communication between hospital cancer specialists and GPs
Rio et al. (2017), Australia [70]	6. Follow-up	73 patients with endometrial cancer Survey (*n* = 31 patients) In-depth phone surveys (*n* = 5 patients) Review of medical records in pre-model cohort group (*n* = 20) GPs of the 73 patients (*n* = 72 GPs) Survey: 37 GPs	To develop a GP model of follow-up care after surgical treatment with early endometrial cancer that provide comprehensive clinical handover to GP and was acceptable for both patients and GPs
Hackett et al. (2018), UK [72]	7. Palliative care	5 focus groups with 27 health professionals (6 GPs) Survey of 24 general practice managers or GP leads Observations of multidisciplinary GSF-meetings in 3 general practices (*n* = 32 health professionals) 8 interviews with healthcare professionals (3 GPs)	To improve understanding of variations in practice using the Gold Standards Framework (GSF: A description of a number of evidence-based principles of practice as a guide for the care of palliative patients and their families) through exploring the perspectives and experiences of members of primary healthcare teams involved in the care of patients with advanced cancer
Finucane et al. (2020), Scotland [71]	Review: 7. Palliative care Interview: 7. Palliative care	Review of 1034 patient journals of deceased patients Cancer patients (*n* = 361) Organ failure (*n* = 265) Frailty and/or dementia (*n* = 408) Interviews with GPs in 17 practices (*n* = 17), and in one practice two nurses was interviewed	To estimate the proportion of people with an advanced progressive illness who had a Key information summary (KIS) by the time of death and when the KIS was started To identify which elements of anticipatory care planning were most frequently recorded in the KIS To explore general practice staff perceptions of the KIS
Non-peer-reviewed case reports			
Larsen et al. (2014), Denmark [43]	2. First presentation and investigation of symptoms 4. Diagnosis 5. Treatment 6. Follow-up 7. Palliative care	50 very ill patients, mostly cancer patients One general practice	To describe experiences of a proactive approach of care towards very ill patients throughout their cancer trajectory
Albinus (2013), Denmark [40]	3. Referral to secondary care 4. Diagnosis 5. Treatment 6. Follow-up 7. Palliative care	Newly diagnosed cancer patients and patients referred with cancer suspicion A general practice (consisting of 3 GPs)	To describe experiences from a general practice with having close contact with patients during the entire cancer trajectory, from referral to secondary care until death or recovery.
NHS (2019), Great Britain [61]	6. Follow-up	Group consultations arranged by one general practice nurse: One group with gynecological cancer patients and similar cancer patients (similar not specified) One group with prostate cancer patients	To describe experiences with a group consultation model for cancer patients in general practice after completion of an accredited education program on group consultations
Hoffmann (2015), Denmark [69]	6. Follow-up 7. Palliative care	General practitioners (GPs) and the departments of urology in Central Denmark Region, with GPs having 0–2 primarily curative treated prostate patients without recurrence and prostate cancer patients with disseminated cancer	To describe experiences from a shared care-project between urology department in hospital and general practice of patients with prostate cancer. When it is possible hospital follow-ups for prostate cancer patients is moved to general practice.
Non-peer-reviewed reports			
Danish Cancer Society (2017), Denmark [37]	2. First presentation and investigation of symptoms 3. Referral to secondary care 5. Treatment	A survey of 5,389 cancer patients	To clarify patient perspectives on the cancer trajectory from first symptoms and contact to the healthcare system, and onwards to completed treatment in secondary care
Danish Cancer Society (2019), Denmark [45]	6. Follow-up 7. Palliative care	A survey of 3,153 cancer patients	To clarify needs and experiences of cancer patients ∼2.5 years after their cancer diagnosis

**Table 3.** Identified healthcare practices that increase the quality of care, their corresponding cancer trajectory phases, and components of quality healthcare.

Healthcare practices that increase quality of care	Cancer trajectory phases	Components of quality healthcare
Listen carefully to the full story	1. Awareness of patients’ bodily sensations and unexplained symptoms 2. First presentation and investigation of symptoms 3. Referral to secondary care 4. Diagnosis 5. Treatment 6. Follow-up 7. Palliation	Efficient quality of care Equitable quality of care People-centered quality of care Timely quality of care
Use action plans and time-out-consultations	1. Awareness of patients’ bodily sensations and unexplained symptoms 2. First presentation and investigation of symptoms 3. Referral to secondary care 4. Diagnosis 5. Treatment 6. Follow-up 7. Palliation	Efficient quality of care Integrated quality of care People-centered quality of care Safe quality of care Timely quality of care
Plan and provide proactive care	4. Diagnosis 5. Treatment 6. Follow-up	Effective quality of care Efficient quality of care Integrated quality care People-centered quality of care Safe quality of care Timely quality of care
Act as an interpreter of diagnosis, treatment options, and its consequences for the patient	4. Diagnosis 5. Treatment 6. Follow-up	Effective quality of care Efficient quality of care People-centered quality of care Safe quality of care
Coordinate care with specialists, patients, and caregivers with mutual respect	4. Diagnosis 5. Treatment 6. Follow-up	Effective quality of care Efficient quality of care Integrated quality of care People-centered quality of care Timely quality of care

### Phase 1. Awareness of bodily sensations and unexplained symptoms

#### II. Communication/decision making

When patients perceive bodily sensations and unexplained symptoms, they might convince themselves that symptoms could be due to age, lifestyle, other known/chronic conditions, or present these to the GP and argue that their symptoms are credible [25–27]. Therefore, patients, caregivers, and GPs are mutually dependent on each other to discuss and negotiate the possible explanations for bodily sensations and unexplained symptoms. *People-centered* and *equitable* quality of care is increased when the GP: listens carefully to the full story, is aware of both verbal and non-verbal communication, and is aware of how their perception of the presented bodily sensations and symptoms may be affected by their previous relationship with the patient or caregivers [26,28].

### Phase 2. First presentation and investigation of symptoms in primary care

#### I. Clinical structure

To provide *effective* and *evidence-based* quality of care, continuous medical education for early cancer diagnostics should be aimed at specific cancer types [29–32]. Time constraints in general practice can be a barrier to *timely*, *efficient*, and *people-centered* healthcare; thus, negatively influencing both the GPs ability to listen to the patients’ full story and their decision to perform a thorough physical examination, and makes it difficult for patients to get an appointment [27,31,33].

#### II. Communication/decision making

The quality of *people-centered* and *equitable* healthcare is increased when GPs: are aware of their preconceptions about their relationship with patients and caregivers who attend the consultation(s), listen to the patients’ full story, and investigates/explores the patients’ preconceptions to avoid miscommunication [28,34–36]. Moreover, *timely* and *effective* quality of care in cancer trajectories is increased if GPs examine the patients’ story for structured, precise, and detailed presentation (e.g. duration of symptom, time course, and associated symptoms) by using close-ended questions to guide the patient’s presentation of their symptoms [33].

#### III. Care coordination

The quality of *timely* and *effective* care related to care coordination is increased, when GPs and patients/caregivers reach an agreement regarding a specific time and date for further follow-up and consultations, which functions as a safety net for both the GP and patients/caregivers [28,35].

#### IV. Patient/caregiver experiences

For most cancer patients, their GP was their first healthcare contact in their cancer trajectories. Thus, *people-centered* quality of care is increased when the GP investigates whether their patients feel that their symptoms are being taken seriously and whether their patients suspect that their symptoms could be cancer-related [31,32,37].

### Phase 3. Referral to secondary care

#### II. Communication/decision making

It increases the quality of *integrated* and *people-centered care*, when GPs provide information and reassurance to patients regarding their referral, based on the patient’s information needs. Furthermore, inquiring about what their patients would like to know about the diagnostic testing process, encompassing referral, specialist input, and how the patient can obtain the results, also increases the quality of care [38]. To increase the *integrated* quality of care, GPs and patients can make an action plan together, which the patient can use in case of delays in the process [39]. An action plan is defined as an explicit and mutual agreement between GPs and patients, where the role of the GP and the responsibilities of both patients and GPs throughout the cancer trajectory are clearly defined.

#### III. Care coordination

Including fast-track referral, proactive care increases *integrated* quality of care, when GPs seek to maintain contact with cancer patients throughout the entire cancer trajectory by systematically making appointments for follow-up consultations [32,40].

### Phase 4. Diagnosis

#### II. Communication/decision making

It increases the *people-centered* quality of care, when GPs act as an interpreter for the patient, by informing the patient in layman’s terms about the diagnosis and its consequences in regards to care, including discussing physical and psychosocial effects [41,42]. It increases the quality of *timely* and *integrated* care when GPs make an action plan [34]. At diagnosis in primary care, it increases the quality of *people-centered* care, if GPs tell patients to bring a relative to their consultation [27].

#### III. Care coordination

To increase the quality of *effective* and *efficient* care, GPs and general practice staff can make checklists of concrete tasks, appoint roles to GPs and practice staff, and describe when and how they should be involved in the cancer trajectory and structured activities. Structured activities that increase *timely*, *people-centered,* and *equitable* quality of care include: sending a letter to patients when the cancer diagnosis is given, describing the potential role of general practice in the cancer trajectory, and then appointing a contact person (i.e. GP or practice staff) to contact the patient if they do not respond to the letter [32,40,43,44].

#### IV. Patient/caregiver experience

It increases the quality of *timely* and *people-centered* care when GPs discuss their role in the cancer trajectory with their patients and caregivers. Due to patients not being informed of the GPs role in the cancer trajectory, there is a risk of them not wanting to bother the GP by contacting them or a risk that previous negative experiences in the cancer trajectory impact their perception of support from their GP [37,44,45].

### Phase 5. Treatment

#### I. Clinical structure

Increasing *effective*, *safe*, *people-centered,* and *integrated* quality of care, requires GPs to maintain contact with patients during treatment phases and hospital admissions, and provide healthcare professionals in the secondary sector (e.g. surgeons, oncologists) with relevant information about the patient [46–48].

#### II. Communication/decision making

If GPs are available to both patients and relatives, to interpret and discuss the diagnosis and its consequences, including physical and psychosocial effects, it increases *effective*, *safe,* and *people-centered* quality of care [44,49–52]. Time-Out-Consultations in general practice are general practice-initiated consultations after diagnosis and before initiation of treatment, aimed at supporting treatment decisions. Moreover, these consultations increase *safe*, *efficient*, and *people-centered* quality of care by supporting patients in choosing treatment, based on both evidence and the patient’s preferences [51,53].

#### III. Care coordination

It increases the quality of *timely* and *people-centered* care when GPs are explicit about the roles of the GP and practice staff in the treatment phase; regarding, the patient’s physical and psychosocial needs related to cancer and other chronic conditions [41–43,47,50,54]. One way to ensure contact is maintained during the treatment phase is to reserve consultation time in the calendar to make room for outreaching patient contact [43]. Having a representative participate in the multi-disciplinary meetings at the hospital on behalf of the patient’s GP, to both provide information and receive information about the patient, increases; *effective*, *people-centered*, and *timely* quality care, especially in complex cases [55]. Additionally, it increases the quality of *people-centered* and *integrated* care if GPs that do not receive adequate information from secondary care, ask them to supply further information [41].

#### IV. Patient/caregiver experiences

Proactive care increases the *timely* and *people-centered* quality of care [32,45,49,52] by helping to identify, among others, patients who distrust their GP due to experiences leading up to their diagnosis, or patients who lack trust in their GPs’ knowledge about their disease [49,50]. Likewise, proactive care may benefit caregivers who might need to be contacted by their GP to discuss physical and psychosocial effects, because they do not contact the GP for fear of wasting the GP’s time [44,54].

### Phase 6. Follow-up

#### I. Clinical structure

GPs’ use of data from electronic patient files for proactive supervision of cancer patients, increases *safe*, *integrated*, *efficient,* and *people-centered* quality of care. Therefore, insufficient exchange of data between general practice and secondary care is a barrier to executing this general practice [36,40,56].

To assure the quality of *timely* care, it is important that GPs ensure that they are accessible to their cancer patients and caregivers so that patients don’t have to wait weeks before getting an appointment [39,44,57,58], e.g. conducting telephone or web consultations with patients and their caregivers [43,59]. Moreover, in cases where patients do not attend follow-up visits, practice staff could contact the patient, inquire why they missed their appointment, and offer them a new consultation [60]. Another approach for delivering *timely* and *efficient* quality of care is to provide follow-up care in a group setting, based on cancer type, and focuses on: addressing illness-specific issues, offering support, reviewing progress, identifying raised needs, and ensuring that previous concerns have been addressed [61].

#### II. Communication/decision making

*Effective* and *people-centered* quality of care during follow-up in the cancer trajectory is increased when GPs act as interpreters of hospital information, such as survivorship care plans and practice proactive care [49,54,56,62,63]. In cases where the patient is familiar with the GP, it increases the *effective* and *people-centered* quality of care if the GP does not completely follow the recommended guidelines regarding exercise and nutrition recommendations based on cancer diagnosis, but instead tailors the recommendations to each individual patient [64]. A well-established relationship before the cancer diagnosis makes it easier for patients, caregivers, and GPs to contact each other, and for patients and caregivers to ask the GP for support [36,44,47]. The patient-GP relationship was strengthened when video consultations were used, if the patient, cancer specialist, GP, and in some cases caregivers, were included [52]. Support and information may also be offered by practice staff or as peer-support in group consultations [43,61].

#### III. Care coordination

It increases quality of *people-centered* and *integrated* care when GPs practice proactive care [32,36,39,41,42,54,57,62,65–67]. Both GPs and practice staff may act as the patient’s contact person in general practice [40], and pre-book appointments for performing outreach patient contact [43].

Only sharing care plans between general practice and secondary care, might not affect patient outcomes [68], since *people-centered*, *integrated,* and *effective* quality of care requires information handed from secondary care to general practice that is both comprehensive and specific for general practice, including; how to manage late effects, which possible symptoms of recurrence to look for, and when the GP should refer the patient back to the specialists [32,41,58,63]. Still, when GPs and specialists show mutual respect and work together (e.g. in video consultation between specialists, GPs, and patients) their sharing of information can increase the *integrated* and *effective* quality of care, with the patient as an obvious part of this teamwork [48,60,69,70]. One way of improving cooperation is for specialists to provide general practice with a direct telephone number in case of questions [69].

#### IV. Patient/caregiver experience

Proactive care increases the quality of *integrated*, *timely,* and *people-centered* care; whereas, follow-up appointments led by GPs improve the relationship between patients and GPs [42,49,54,57]. Proactive care requires the GP to be: friendly, have knowledge about the disease, appear receptive to questions, and be explicit about the process [39]. *People-centered* quality of care is increased, when GPs ask for patients’ perceptions of their relationship, which might be affected by experiences from consultations before diagnosis and patients’ perceptions of the GP’s cancer-related knowledge [42,45,49,63]. Furthermore, the quality of *people-centered* and *timely* care increases when GPs proactively care for relatives and caregivers during follow-up [32,44,54].

### Phase 7. Palliative care

#### I. Clinical structure

*Effective*, *safe*, *integrated,* and *people-centered* quality of care increases when GPs ensure they have information regarding preferred place of death, medical condition, information about the living situation, names and telephone numbers of family, and a known treatment plan with specified responsibilities of multidisciplinary health professionals, including appointing a stand-in for the regular GP if the GP is absent. The quality of *people-centered* care is increased if the GP performs home visits, and offers GP consultations to caregivers [43,44,71,72]. Moreover, *effective* quality of care increases when GPs participate in continuous medical education, especially if they lack palliative care knowledge [73].

#### II. Communication/decision making

Quality of *people-centered* and *integrated* care is increased, if GPs initiate and negotiate their involvement in palliative care [72,74] and use dialogue to identify needs, even in cases when there is no clear prognosis [46,59,74].

#### III. Care coordination

To increase *effective*, *integrated*, *timely,* and *people-centered* quality of care, besides being responsible for prescriptions and follow-up of already identified needs, GPs can ensure early identification of palliative care needs [46,74]. Home visits and telephone or video consultations between the patient, GP, and secondary care, increase the quality of *integrated*, *effective,* and *people-centered* care by; managing pain, managing comorbidity, providing psychosocial support, and building-up relationships [48,52,59,72]. Moreover, communication from secondary care regarding; clinical information, information about patient and family, living situation, and preferred place of death, are very useful in general practice [71]. In regards to managing a patient’s acute needs, the quality of *timely* care increases if patients have the GP’s direct number [40].

#### IV. Patient/caregiver experience

Performing Time-Out-Consultations with patients in the palliative phase increases the *timely* and *people-centered* quality of care [46,75].

## Discussion

### Principal findings

A total of 51 studies that presented healthcare practices in cancer trajectories from a general practice perspective were identified. These studies provided knowledge of how healthcare practices increase the quality of care in the different phases of cancer trajectories. This scoping review reflects the context of primary care: balancing increased demands for efficiency, greater complexity of biomedical knowledge, and consideration for individual patient needs [76]. Overall, this study found that it increases the quality of care in all cancer trajectory phases when GPs; (1) Listen carefully *to the full story* and (2) *Use action plans*. After referral for secondary care, quality of care is increased when GPs and practice staff: (I) *Use a proactive care approach*, (II) *Act as interpreters of diagnosis, treatment options, and its consequences*, and (III) *Engage in care coordination with specialists in secondary care involving the patient*. While *time constraints and accessibility to general practice* can be substantial barriers to quality of care.

### Findings in relation to other studies

#### Listen carefully to the full story

A review described the importance of listening to the full story, emphasized by the GPs’ use of their ‘gut feeling’ when listening to patients’ descriptions of symptoms and non-verbal cues during the diagnostic phase [77]. However, GPs’ listening to their ‘gut feeling’ is related to the GPs’ perceived relationship with the patient [78], and is concurrent with our finding that GPs should be aware of their preconceptions about the relationship. Moreover, a study concluded that diagnosing cancer is not solely a question of adhering to clinical guidelines [79]. The suspicion of a cancer diagnosis arises during GP and patient communication, and ineffective communication can cause a delay in a timely cancer diagnosis [80].

During treatment and follow-up care, the use of self-reported needs assessment questionnaires completed at home before a general practice consultation supports patients with cancer to reflect and articulate their own perception of problems and needs [81]. Such a tool in general practice could support the patient in presenting ‘the full story’.

#### Use action plans

The use of action plans is described in all phases of the cancer trajectory. A review exploring the role of GPs’ ‘gut feelings’ in the diagnostic phase, reported that GPs encouraged patients to contact the GP again if their symptoms persisted or worsened [77]. This study found that the GP should do more than just encourage patients to re-consult. Based on the GPs evaluation of each patient, as to whether the patient will contact the GP if their symptoms persist, the GP either scheduled a follow-up consultation or relied on the patient to contact the GP in case of persistent or worsening symptoms. Booking the next follow-up visit at the end of the consultation will avoid hindering patients from booking a GP consultation due to waiting time. Likewise, planning a follow-up consultation gives GPs with perceived time pressure, time for; a more thorough physical examination, eliciting clinical signs, and listening to the patient’s full story [80].

One study described GPs’ opinions about using text messages to communicate with patients with low-risk cancer symptoms. The study found that text messages could act as a safety net by encouraging patients to either remember their consultation or encouraging patients to contact their GP if the patient’s symptoms persist or worsen [82]. Text messaging is already being used in general practice, but to the knowledge of the authors, no existing literature describes the systematic use of text messages and the effects thereof.

Another positive aspect of having an action plan is that it addresses the patient’s need for knowing whom to contact in case of emerging problems and needs. This study found that after patients are diagnosed with cancer, they often do not have information regarding what role their GP plays in the patient’s cancer trajectory; however, this can be remedied by coordinating care, such as telephone and video consultations between specialists, patients, and GPs [59,83]. Moreover, it is also important for the GP to be informed of the action plans and responsibilities the specialists and patients agreed upon [84].

#### Use a proactive care approach

As this study found, a proactive care approach supports those patients who are in-between hospital departments during the diagnosis and treatment phases and do not know who to contact if problems or needs emerge [84]. Furthermore, this approach supports patients who distrust their GP due to previous negative experiences [84] or lack of confidence in their GP’s knowledge of their cancer diagnosis, treatment, and follow-up [85,86].

In the palliative phase, WHO recommends early identification of palliative care needs by GPs [23], and WHO has developed eight indicators that GPs may use for needs identification [87]. However, further investigation of the effect of these is required.

#### Act as interpreters of diagnosis and its consequences

In accordance with the results of this study, a review found that men diagnosed with prostate cancer are at risk of regretting their treatment decisions. GPs can support these patients by encouraging them to include their personal values and level of social support in the decision-making process before treatment; furthermore, GPs can review and interpret treatment information material together with the patient [88].

Another review found that the major barrier for using the GP’s cancer-related knowledge after diagnosis is GPs’, patients’ and specialists’ lack of trust in the GPs’ knowledge [85]; thereby, limiting the GPs ability to provide adequate information and timely identification of needs and symptoms that require referral to secondary care. Another study found that, if GPs had received additional training and could contact a specialist in case of questions, patients trusted the GPs to be responsible for their follow-up care; even though, all tasks in follow-up care may not be identified in advance [84].

#### Engage in care coordination with specialists in secondary care involving the patient

Care coordination should not be the responsibility of either specialists or GPs, but both [89]. However, shared information and the organization of follow-up care should not be standardized or solely based on organizational and administrative decisions [90]. The decision as to whether follow-up cancer care should be shared between GPs and specialists, or whether follow-up care should only be provided in either primary care or a hospital setting, could be a risk-stratification of cancer patients [91] based on cancer-related effects, comorbidity, and socioeconomic disparities [92]. Finally, care coordination should include the clearly defined roles of both the specialist and the GP, how the specialist and GP can contact one another, and a plan or guideline for follow-up care provided to the GP by the specialist [85,93].

One method for improving care coordination is by using shared care plans, most often developed in secondary care, then sent to both the patient and the patient’s GP [94]. However, content requirements for shared care plans differ for GPs and patients. One study found that primary care providers were more interested in receiving information about the late effects of treatment, rather than specifics regarding therapeutic agents and dosage [95]; additionally, the study found that patients wanted a plan that described what they could expect throughout their cancer trajectory. Moreover, the same study described that specialists’ wished for an ‘interactive’ document, that could be continuously updated [95]. Even though there are many wishes for shared care plans, the effect of care plans for patients remains unclear. A randomized controlled trial found that implementing a shared care plan increased patients’ concerns, symptoms, and contact to their GP with cancer-related concerns [96]. Most importantly, the mere presence of care plans does not imply improved coordinated care, unless they are implemented as a tool to support communication and shared care for specialists, GPs, and patients [97].

### Strengths and weaknesses of the study

The findings of this scoping review should be considered along with some limitations. Even though a robust search for literature assisted by a research librarian was executed, it’s possible some relevant literature (including literature written in languages other than English and Danish) was missed. Further, it’s recognized that healthcare systems vary greatly across countries, and even though this study included international literature, the evidence presented in this review may not be universally applicable or actionable. Furthermore, the findings may not apply to all cancer types, as included studies predominantly focused on patients with breast, prostate, and/or colorectal cancer.

The scoping review method used in this study is a strength, as it enabled the investigation of research in the context of general practice, and presented results integrating the complexity of quality of care. However, a limitation of scoping reviews is that the included studies are not quality-rated. Nonetheless, the aim was to identify the variation of healthcare practices increasing quality of care, not to identify correct healthcare practices, since correct healthcare practices vary due to the complexity of quality of care in general practice.

### Implications for clinicians

This scoping review identified healthcare practices that increase the quality of care in cancer trajectories from a general practice perspective. Even though some of the identified healthcare practices are already being implemented in many general practices, the results may help to further guide individual GPs and general practice teams to organize and address quality cancer care by using the results of this scoping review to identify areas they can initiate quality improvement initiatives, and adjust their healthcare practices according to the increasing demands of efficiency, greater complexity of biomedical knowledge, and consideration for individual patient needs. Moreover, this scoping review informs; general practice, hospital specialists, policymakers, and interest organizations on how to improve the quality of care in cancer trajectories.

## Appendix I

**Table A.1.** Example of search in Medline using a combination of three search blocks.

Search block	Search terms
1	Cancer* OR neoplasm*
2	Patient pathway* OR continuity of patient care OR shared care OR co-manag* OR patient care manag* OR collaborative care OR care coordinat* OR continuum OR proactive care OR relationship centered care OR patient-focused care OR patient-centered nursing OR patient-centered communication OR shared decision* OR physician-patient relation* OR nurse-patient relation* OR attitude of health personnel OR attitude of patient* OR communication OR professional competence* OR patient experience* OR caregiver experience*
3	General practice OR general practice staff OR general practitioner* OR primary care physician* OR patient care team OR family physician OR primary care practitioner* OR family practice*
